# REtinal Detachment Outcomes Study (REDOS): study protocol for a factorial, randomized controlled trial

**DOI:** 10.1186/s13063-023-07815-x

**Published:** 2023-12-20

**Authors:** Mélanie Hébert, Serge Bourgault, Mathieu Caissie, Éric Tourville, Ali Dirani

**Affiliations:** Department of Ophthalmology, Hôpital du Saint-Sacrement, CHU de Québec – Université Laval, 1050 Ch Ste-Foy Street, Québec, QC G1S 4L8 Canada

**Keywords:** Pars plana vitrectomy, Scleral buckle, Rhegmatogenous retinal detachment, Proliferative vitreoretinopathy, Anatomic success, Visual acuity, Retinal displacement, Postoperative pain, Quality of life, Complications

## Abstract

**Background:**

Few large randomized controlled trials provide strong evidence to guide surgical repair of primary rhegmatogenous retinal detachment (RRD) repair. The purpose of this factorial, single-blind, randomized controlled trial is to analyze and compare the surgical outcomes, functional visual outcomes, complications, and quality of life associated with RRD repair using (A) pars plana vitrectomy only (PPV) or PPV with scleral buckle (PPV-SB) and (B) sulfur hexafluoride gas (SF_6_) or perfluoropropane gas (C_3_F_8_) tamponade.

**Methods:**

Eligible patients with moderately complex RRD will be randomized 1:1 to PPV or PPV-SB and 1:1 to SF_6_ or C_3_F_8_ gas tamponade. Approximately 560 patients will be recruited to be able to detect a difference of around 10% in SSAS rate between the groups. Patients will be followed using multimodal imaging and quality of life questionnaires after the surgical repair until 1 year postoperative. The primary outcome will be a single-surgery anatomic success (SSAS), defined as the absence of reoperation for recurrent RRD in the operating room. Secondary outcomes will be pinhole visual acuity (PHVA) at 8–10 weeks and 6 months, final best-corrected visual acuity (BCVA), final retina status (i.e., attached or detached), time to onset of RRD recurrence, severity and number of complications, and questionnaire results.

**Discussion:**

This will be the first 2 × 2 factorial RCT examining repair techniques in primary RRD. It will also be the first RCT to compare gas tamponade between the two most common agents. Notably, it will be adequately powered to detect a clinically significant effect size. The use of multimodal imaging will also be a novel aspect of this study, allowing us to compare head-to-head the impact of adding an SB to the retina’s recovery after RRD repair and of differing gas tamponades. Until now, the treatment of RRD has been largely guided by pragmatic retrospective cohort studies. There is a lack of strong evidence guiding therapeutic decisions and this trial will address (1) whether supplemental SB is justified and (2) whether longer duration gas tamponade with C_3_F_8_ is necessary.

**Trial registration:**

ClinicalTrials.gov NCT05863312. Registered on 18 May 2023.

**Supplementary Information:**

The online version contains supplementary material available at 10.1186/s13063-023-07815-x.

## Administrative information

**Table:** 

**Title {1}**	**REtinal Detachment Outcomes Study (REDOS)**
Trial registration {2a and 2b}	ClinicalTrials.org NCT05863312
Protocol version {3}	2023–10-31 Version 4
Funding {4}	Fighting Blindness Canada Clinician-Scientist Emerging Leader Award funding
Author details {5a}	Mélanie Hébert, Hôpital du Saint-Sacrement, Centre hospitalier universitaire de Québec – Université Laval Serge Bourgault, Hôpital du Saint-Sacrement, Centre hospitalier universitaire de Québec – Université Laval Mathieu Caissie, Hôpital du Saint-Sacrement, Centre hospitalier universitaire de Québec – Université Laval Éric Tourville, Hôpital du Saint-Sacrement, Centre hospitalier universitaire de Québec – Université Laval Ali Dirani, Hôpital du Saint-Sacrement, Centre hospitalier universitaire de Québec – Université Laval
Name and contact information for the trial sponsor {5b}	CHU de Québec – Université Laval, Research Ethics Board Email: gurecherche@chudequebec.ca Phone: + 1 418 525–4444 #52715
Role of sponsor {5c}	This is an investigator-initiated study. The funder (Fighting Blindness Canada) is not involved in and does not have authority over any aspects of this study, including study design, data collection, management, analysis, interpretation, report writing, and publication. The sponsor (CHU de Québec – Université Laval) has ethical and legal oversight over the proper conduct of research by ethical guidelines but no input on any aspects of this study

## Introduction

### Background and rationale {6a}

Two important and common techniques used in the surgical repair of rhegmatogenous retinal detachment (RRD) are pars plana vitrectomy (PPV) and scleral buckle (SB). Both can be used alone or in combination with a gradual shift towards PPV with improvements in technology and technique. Prominent retrospective cohort studies such as the Primary Retinal Detachment Outcomes (PRO) study suggest that a combination of PPV and SB (PPV-SB) has an advantage over PPV alone for phakic and pseudophakic patients [1, 2]. Similarly, a meta-analysis of cohort studies suggests that there may be an increased rate of surgical success without reoperation in patients receiving PPV-SB [3]. However, the few randomized controlled trials (RCT) with small sample sizes (range 30 to 100 patients per group) and heterogenous surgical techniques looking into this question have not found an advantage to additional SB [4–7], and a recent meta-analysis of RCT has also not found an advantage but rather increased myopization, duration of surgery, and postoperative pain following additional SB [8]. In a way to simulate randomization, we performed a propensity score analysis comparing the surgical outcomes of PPV compared to PPV-SB at our center which also showed similar results between both techniques [9]. The discrepancy between the results of non-randomized and randomized studies in this debate is likely due to indication bias, that is, the tendency of surgeons to treat more complex patients using PPV-SB thinking that it will provide them with a better outcome.

In addition to this choice of surgical technique, there is also the use of different gas tamponades which can be of interest. Most commonly, vitreoretinal surgeons use either sulfur hexafluoride gas (SF_6_) or perfluoropropane gas (C_3_F_8_) to maintain the reapproximation of the retina postoperatively, reserving the use of silicone oil to refractory cases where a longer tamponade is needed. SF_6_ remains in the eye for 2 to 3 weeks, while C_3_F_8_ remains in the eye for 6 to 8 weeks. There may therefore be an indication bias to use C_3_F_8_ in more complex cases where a longer tamponade duration is desired. Unfortunately, there are no RCT comparing these two tamponade agents, while studies comparing air and gas tamponades have had mixed results [10, 11].

### Objectives {7}

The objective of this study is to analyze and compare the surgical outcomes, functional visual outcomes, complications, and quality of life associated with primary RRD repair using (A) PPV or PPV-SB (primary objective) and (B) SF_6_ and C_3_F_8_ gas tamponade (secondary objective). Our working hypotheses are as follows:

1. PPV and PPV-SB have similar single-surgery anatomic success (SSAS) rate.
2. PPV may lead to better final best-corrected visual acuity (BCVA), less complications, and better quality of life compared to PPV-SB.
3. SF_6_ and C_3_F_8_ gas tamponades have similar SSAS rates, visual outcomes, complications, and quality of life.
4. There is minimal to no significant interaction between surgical technique (i.e., PPV or PPV-SB) and gas tamponade (i.e., SF_6_ and C_3_F_8_).

### Trial design {8}

This is a large, 2 × 2 factorial, interventional, randomized controlled trial. Eligible patients with primary RRD will be randomized using a simple randomization 1:1 to receive PPV or PPV-SB and SF_6_ or C_3_F_8_ gas tamponade. Statistical analyses of the outcomes will aim to demonstrate the superiority of one technique over the other.

## Methods: participants, interventions and outcomes

### Study setting {9}

The study will take place at the Centre Hospitalier Universitaire de Québec – Université Laval, a tertiary care, academic center in Québec City, Canada.

### Eligibility criteria {10}

Patients aged ≥ 18 years diagnosed with RRD will be considered for inclusion. Since very simple RRD are more likely to be treated with PPV, while more complex RRD are more likely to be treated with PPV-SB, we will select patients who have a moderately complex RRD, like the PRO study. This excludes the following characteristics: proliferative vitreoretinopathy (PVR) grade ≥ C2, chronic RRD with duration > 3 months, proliferative diabetic retinopathy with tractional retinal detachment (RD), macular holes, epiretinal membrane grade 3 or 4, traumatic RD, giant retinal tears, retinal dialysis, foveoschisis, wet age-related macular degeneration, endophthalmitis, acute retinal necrosis, Coats disease, retinopathy of prematurity, retinoschisis, retinal colobomas, prior glaucoma surgery or strabismus surgery (difficulty with adding SB), and superior RD extent less than 3 clock hours (favoring PPV).

Age younger than 45 years is a relative exclusion factor whereby, if the surgeon considers the patient more eligible for an SB-only procedure, they will not be considered for the study. In contrast to the PRO study, we will accept moderate degrees of PVR and RD extent greater than 9 clock hours (favoring PPV-SB). We also did not consider certain factors which were included given that the PRO study also included SB-only procedures (e.g., significant vitreous opacities, cataract, prior vitrectomy, greater than moderate vitreous hemorrhage).

### Who will take informed consent? {26a}

Designated research team members will be tasked with explaining trial details and obtaining written informed consent from eligible patients after being presented with the study by members of the clinical care team.

### Additional consent provisions for collection and use of participant data and biological specimens {26b}

Vitreous samples could be collected as part of a biobank at the center for which a separate, parallel consent process will be used.

### Interventions

#### Explanation for the choice of comparators {6b}

Currently, PPV and PPV-SB are both commonly used procedures for the repair of simple RRD and differences differ between centers and practitioners. The use of SF_6_ or C_3_F_8_ gas tamponades is also often left to the surgeon’s discretion. Both procedures will therefore be compared in this trial. The current standard of care involves the operating surgeon choosing whether to place an SB or not, as well as whether to place SF_6_ or C_3_F_8_ according to their personal preference. This is only guided by expert opinion at the moment in the absence of high-grade evidence; hence, this justifies the relevance of this study to better support decisions in practice.

#### Intervention description {11a}

Surgery will be performed under local anesthesia with retrobulbar block (5 to 10 cc of 2% lidocaine without epinephrine and 0.5% bupivacaine in a 1:1 ratio). In all cases, surgery will be performed using a wide-angle viewing system and the Alcon Constellation System (Alcon Laboratories, Inc., Fort Worth, TX) combined with the ULTRAVIT 23-G + or 25-G + vitreous cutter (Alcon).

Pars plana vitrectomy will be performed in a standard fashion starting with central vitrectomy, then by localizing retinal breaks and marking them with endodiathermy. Perfluorocarbon will be used to displace subretinal fluid which will be aspirated at its exit from the retinal break as much as possible to avoid retinal pigment epithelium (RPE) dispersion into the vitreous and maximal vitreous base shaving will be performed in all cases. This will be followed by an air-fluid exchange. The use of cryotherapy to solidify the retina intraoperatively and the use of internal limiting membrane peeling of the posterior pole will be at the discretion of the surgeon. In all cases, laser photocoagulation around retinal breaks, holes, areas of lattice degeneration, and posterior to sclerotomy sites will be done and then a 360° laser retinopexy will be performed at the surgeon’s discretion and consisted of three rows of medium-white burns anterior to the level of the vortex vein, towards and beyond the equator. In cases with SB, after 360° peritomy and dissection in 4 quadrants, a 41-circling band with 3082 sleeves (Labtician Ophthalmics, Oakville, ON, Canada) will be used in all cases and fixed to the sclera at approximately 11.5 mm from the limbus (or 5.5 from the insertion of rectus muscles) using partial thickness scleral tunnel or mattress sutures with 5.0 prolene or nylon performed in four quadrants depending on the surgeon preferences. At the end of the surgery, a gas tamponade will be injected inside the eye, either SF_6_ or C_3_F_8_. The surgical technique at the second surgery, if applicable, will be at the discretion of the treating surgeon.

#### Criteria for discontinuing or modifying allocated interventions {11b}

Patients who would develop severe complications from their SB (e.g., implant infection or intractable pain) which would require removal of the SB could undergo a second operation to remove it. Should the patient require silicon oil as a tamponade agent as deemed by the treating surgeon upon starting the operation (e.g., in cases of extensive PVR undetected preoperatively), the patient could therefore be removed from the study and treated accordingly.

#### Strategies to improve adherence to interventions {11c}

The intervention allocation will be determined based on the preoperative appearance of the RRD and will not be permitted to change once in the operating room.

#### Relevant concomitant care permitted or prohibited during the trial {11d}

Any concomitant care required by the patient’s ophthalmological condition will be permitted during the trial, including other medical treatments or surgical procedures.

#### Provisions for post-trial care {30}

After the 1-year follow-up included as part of the trial, if there are still ongoing retinal or other ophthalmological conditions which require active subspecialty management, patients will continue to be followed by the retina or ophthalmology team of the CHU de Québec – Université Laval indefinitely or until resolution of said conditions. If there are no ongoing retinal or other ophthalmological conditions, standard eyecare follow-up could be performed by community optometrists.

### Outcomes {12}

The primary outcome is SSAS defined as freedom from reoperation for recurrent RRD at all follow-up evaluations. Localized recurrences or new tears treated using a laser in the ambulatory clinic will not be considered a failure of the primary surgery but will be noted. We considered a difference of 10% in SSAS to be clinically relevant for this trial and based the sample size calculation on this threshold of chosen efficacy. Secondary outcomes will include PHVA at 8–10 weeks and 6 months, final BCVA, final retina status (i.e., attached or detached), time to onset of RD recurrence, severity and number of complications, and questionnaire results (see Additional file 1). The principal questionnaire was previously used and validated by Potic et al. [12] with additional questions used to specifically assess possible side effects and reductions in quality-of-life associated with the addition of SB and longer-duration intraocular gases. In cases of primary surgery failure, the cause of recurrence (i.e., PVR including epiretinal membrane (ERM) in the macular area causing recurrent RD, new or recurrent retinal breaks) will be reviewed. The extent of PVR at follow-up will also be evaluated. The duration of surgery will also be collected. Number of additional visits and supplementary laser and intraocular pressure treatments will be evaluated. Complications will be graded using the novel Classification of Ophthalmological Complications (COC; see Table 1).

**Table 1:** Classification of ophthalmological complications (COC)

Complication grade	Definition
Grade I	Any deviation from the normal postoperative course without the need for modification of planned pharmacological treatment or surgical intervention Allowed therapeutic regimens are as follows: any previous medication, any medication prescribed routinely (i.e., topical antibiotics, glaucoma drops, anti-inflammatory drops, steroid drops, oral pills), artificial tears, hot compresses, and analgesic drugs (Advil/Tylenol only)
Grade II	Requiring change in planned pharmacological treatment with higher frequency or longer duration of drops or the introduction of new drugs (including autologous serum drops); these can include glaucoma drops and steroid drops and the use of oral drugs other than Advil/Tylenol (e.g., Diamox, prednisone). Also includes YAG capsulotomy
Grade III	Requiring any laser procedures other than YAG capsulotomy or any periocular/intraocular injections. Simple interventions performed at the slit lamp (e.g., AC burp or tap, AC filling, epithelial debridement) or under local anesthesia not requiring an OR setup (e.g., pneumatic retinopexy, keratectomy, leaking wound requiring additional suture) are also included
Grade IV	Requiring intervention with an OR setup or requiring pharmacological treatment with intravenous drugs or hospitalization
Grade V	Sight-threatening complication or loss of eye (endophthalmitis, globe rupture, suprachoroidal hemorrhage)
Grade Va	Unilateral sight-threatening complication or loss of eye (endophthalmitis)
Grade Vb	Bilateral sight-threatening complication or loss of eyes (bilateral endophthalmitis, bilateral eye trauma, sympathetic ophthalmia)
Grade VI	Life-threatening complications (including CNS complications) requiring IC/ICU management
Grade VII	Death

### Participant timeline {13}

Table 2 illustrates the patient timeline for the study. Preoperative baseline characteristics will include age, sex, symptoms duration, pinhole visual acuity (PHVA) at presentation in metric Snellen notation defined as the best visual acuity obtained using the patient’s current refraction with or without improvement with pinhole, laterality of presentation, myopia greater than four diopters, lens status (i.e., aphakic: patient without their native crystalline lens or an intraocular lens; phakic: patient with their native crystalline lens; pseudophakic: patient with an intraocular lens replacing their native crystalline lens), macula status (i.e., on, off, split), RD extent in clock hours, number of retinal breaks assessed preoperatively and under direct intraoperative visualization, and inferior retinal breaks in the detached retina between 4:00 and 8:00 clock hours. Patients will be assessed at 1 day postoperatively, 2 weeks, 8–10 weeks, 6 months, and 12 months and any additional follow-ups as needed on an emergent basis. At each scheduled follow-up, visual acuity will be assessed using a Snellen visual chart with pinhole and intraocular pressure measured by Goldmann applanation, iCare, Accupen, and Tonopen. LOCS III grading for cataract development will be used to evaluate patients’ lens at each follow-up. Multimodal imaging using macular optical coherence tomography (OCT), optic nerve/retinal nerve fiber layer (RNFL) OCT, ganglion cell layer (GCL) OCT measures, OCT angiography, widefield fundus photography, and fundus autofluorescence will be conducted when it is possible (after gas disappearance at 6 months and 12 months). Starting at the 8–10-week follow-up, autorefraction will be used. Quality of life questionnaires will also be administered to patients at each follow-up starting at the 2-week postoperative follow-up.

**Table 2:**
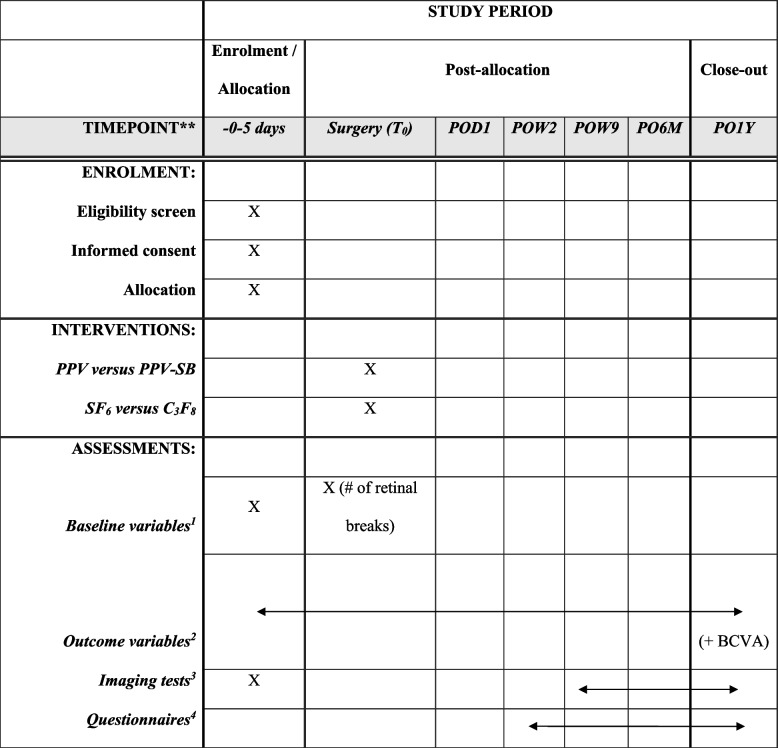
Schedule of enrollment, interventions, and assessments in the REDOS trial

^1^Age, sex, symptoms duration, and pinhole visual acuity (PHVA) at presentation in metric Snellen notation defined as the best visual acuity obtained using the patient’s current refraction with or without improvement with pinhole, laterality of presentation, myopia greater than 4 diopters, lens status (i.e., aphakic, phakic, pseudophakic), macula status (i.e., on, off, split), RD extent in clock hours, number of retinal breaks assessed preoperatively and under direct intraoperative visualization, and inferior retinal breaks in the detached retina between 4:00 and 8:00 clock hours

^2^PHVA intraocular pressure measured by Goldmann applanation, iCare, Accupen, and Tonopen; LOCS III grading for cataract development; retina status (i.e., attached or detached)

^3^Multimodal imaging using macular optical coherence tomography (OCT), optic nerve/retinal nerve fiber layer (RNFL) OCT, ganglion cell layer (GCL) OCT measures, OCT angiography, widefield fundus photography, and fundus autofluorescence will be conducted when it is possible (e.g., after gas disappearance at 6 months and 12 months). Starting at the 8–10-week follow-up, autorefraction will be used

^4^See Additional file 1

The 2-week follow-up examination could be performed on select patients outside of the study center. These include patients from remote regions outside Quebec City and those from regions prohibiting travel to the study center with intraocular gas tamponade (e.g., through mountainous or high-altitude regions). In those cases, the postoperative questionnaire would be answered with a research team member by phone.

Patients with macula-on RRD will be operated on within 48 h of preoperative assessment and allocation, while patients with macula-off RRD will be operated on within 5 days of preoperative assessment and allocation.

### Sample size {14}

We based our sample size calculations on the size necessary to detect a clinically significant effect in surgical technique, given that estimates of effect size among gas tamponades are not as precise in the literature and are a secondary purpose of our study. Based on previous results from our center, SSAS is achieved in 92% and 88% of phakic patients undergoing PPV and PPV-SB, respectively, compared to 91% and 90% in pseudophakic patients. We therefore have at most a 4% difference between the treatment groups. In the PRO study reports, SSAS rates differed between PPV and PPV-SB with 83% and 91%, respectively, in phakic patients and 84% and 92% in pseudophakic patients. Based on our previous results, this would yield a small effect size of about 0.13, while this would be approximately 0.25 according to the treatment differences in the PRO study reports. Considering that a treatment difference smaller than what was found in the PRO study could be deemed not clinically significant, we are proposing a total sample size of 560 patients (*n* = 140 per group) which, assuming a 10% drop-off rate, would still allow us to detect an effect size of 0.25 with a power greater than 80% (see Table 3).

**Table 3:** Total sample size calculation to detect an effect in the ranges of 0.20 to 0.30 for surgical technique (i.e., PPV and PPV-SB) at various study powers. We are proposing a sample size of 560 patients. Corresponding effect size and power will be achieved for the analysis of gas tamponades (i.e., SF_6_ and C_3_F_8_)

Effect size	Power		
	80%	85%	90%
0.20	784	900	1052
0.25	**504**	**576**	672
0.30	348	400	468

### Recruitment {15}

The clinical team will be responsible for identifying patients with RRD and first presenting the study to them. A dedicated research assistant will then be responsible for screening all patients with RRD presenting to the retina team of the CHU de Québec – Université Laval. When an eligible patient is identified, they will be explained in full the option of participating in the trial and signed consent will be obtained. Given that approximately 350 RRD cases are operated every year at the center, up to 4–5 years of recruitment are anticipated for this study.

### Assignment of interventions: allocation

#### Sequence generation {16a}

Given the large sample size that we aim to achieve, patients will be randomized using simple randomization with computer-generated random numbers at the time of enrollment. A sequence will therefore not be generated in advance. No stratification will be used given that the goal is to allow for the generalizability of the results to any patient presenting with moderately complex RRD.

#### Concealment mechanism {16b}

Because each patient will be allocated to their intervention by simple randomization, a concealment of intervention sequence is not required.

#### Implementation {16c}

The simple randomization will be done at the time of enrollment by the research assistant who will obtain the signed patient consent.

### Assignment of interventions: blinding

#### Who will be blinded {17a}

Outcome assessors, investigators, and data analysts will be blinded to the assignment of the interventions and the attributed code in the database until the end. For feasibility reasons, the trial participants and the clinical team including the treating surgeon will not be blinded to the intervention allocation. Blinding of the treating surgeon in relation to surgery technique will not be possible given that they will be responsible for adding the SB, and this will be visible on dilated fundus examination at follow-up given the scleral depression caused by the SB, while blinding to gas tamponade will not be feasible given that the gas bubble will dissipate at a faster rate with SF_6_ compared to C_3_F_8_ on follow-up examinations. As for trial participants, they are operated under local anesthesia with a retrobulbar block and sedation. They can therefore overhear surgeons discussing the next steps with the scrub nurse, deduce the addition of the SB based on duration of surgery, and see the speed at which the gas disappears from their visual field. 

#### Procedure for unblinding if needed {17b}

Patients who would develop severe complications from their SB (e.g., implant infection or intractable pain) which would require removal of the SB could undergo a second operation to remove it. This would be managed with the clinical team which is also not included in the blinding.

### Data collection and management

#### Plans for assessment and collection of outcomes {18a}

Trained and designated research team members will be responsible for collecting baseline and outcomes data with standardized methods of PHVA measurement and questionnaires. A training session of an hour will be provided to each research team member by a single investigator (MH) to promote data quality.

Regarding the intraoperative description of the RRD, a standardized RRD reporting form will be filled out by each treating surgeon at the time of the operation.

#### Plans to promote participant retention and complete follow-up {18b}

Follow-ups will be scheduled and organized by the research team and regular reminders will be performed to reduce the risk of losses to follow-up. Should a participant discontinue the study follow-ups, the reason for discontinuation, as well as the final BCVA and status of the retina (i.e., attached or detached), will be obtained from an eyecare professional before closing the participant’s study file.

#### Data management {19}

Trained and designated research team members will be responsible for entering research data which will be stored in a centralized, secure REDCap server [13, 14] at the CHU de Québec – Université Laval. The case report form will include range checks for data values and standardized input values for dates. A training session of an hour will be provided to each research team member by a single investigator (MH) to promote data quality. Each patient will be coded in the dataset to preserve confidentiality, and the key will only be available to the principal investigator (AD).

#### Confidentiality {27}

All research files including signed patient consents will be kept under lock and key in the ophthalmology clinical research department of the CHU de Québec – Université Laval and will only be available to authorized research team members. Trial participants will be assigned a code in the dataset to preserve confidentiality and the key will only be available to the principal investigator (AD). Nominal data will be destroyed 15 years after the conclusion of the study.

#### Plans for collection, laboratory evaluation and storage of biological specimens for genetic or molecular analysis in this trial/future use {33}

Samples of undiluted and diluted vitreous could be collected at the start of surgery as part of an ongoing biobank (Research Ethics Board number: 2021–5991) at our center independent of the current study. These would be transferred on ice to the laboratory where they will be stored in a dedicated refrigerator. Cytokine profiling could be performed on the samples using multiplex array kits as well as RPE cell quantification.

### Statistical methods

#### Statistical methods for primary and secondary outcomes {20a}

The primary outcome will be compared between the groups using the chi-square test for the difference in rates of SSAS in both the surgical technique and gas tamponade arms of the study. Testing between the groups for secondary outcomes of PHVA at 8–10 weeks and 6 months, final BCVA, final retina status (i.e., attached or detached), time to onset of RD recurrence, severity and number of complications, and questionnaire results will also be performed using adjustments for repeated measures and multiple comparisons as appropriate.

To calculate estimates of the treatment effects and their corresponding 95% confidence intervals (CI), a multiple logistic regression including both surgical technique and gas tamponade will be built for SSAS. Cox proportional hazards and multiple linear regression models will also be built for the time to onset of RD recurrence and final BCVA, respectively. In these analyses, we assume that there is a negligible interaction between surgical technique and gas tamponade. However, appropriate interaction terms will be added to the models to explore this possibility. If this shows negligible interaction, both analyses will be performed independently.

Statistical analyses will be performed using R for Windows (version 3.6.3; R Foundation for Statistical Computing) and IBM SPSS Statistics for Windows (version 27.0; IBM Corp., Armonk, NY). Analyses will be conducted at the 0.05 significance level, except when appropriate for adjustment of multiple comparisons.

#### Interim analyses {21b}

An interim analysis will be conducted in the middle of trial recruitment to verify that no statistically significant difference in SSAS is detectable. Should the difference be significant (> 10% between the groups) and place the patients in one of the groups at increased risk if recruitment is continued, the principal investigator (AD) reserves the right to make the final decision to terminate the trial.

#### Methods for additional analyses (e.g., subgroup analyses) {20b}

Subgroup analyses that are envisioned at the conclusion of this study will include exploratory analyses for outcomes among patients by lens status (i.e., aphakic, phakic, pseudophakic), by macula status (i.e., on, off, split), and by the presence of an inferior RD. Adjustments for multiple comparisons will not be performed for these subsequent analyses.

#### Methods in analysis to handle protocol non-adherence and any statistical methods to handle missing data {20c}

Outcomes will be analyzed as randomized. Missing data will be handled using multiple imputation.

#### Plans to give access to the full protocol, participant-level data and statistical code {31c}

The full protocol will be published in the journal *Trials*. The final trial dataset could be made available to other research teams upon reasonable request and after evaluation of the request by the trial team. The shared dataset will be coded and will not include any identifying patient data.

### Oversight and monitoring

#### Composition of the coordinating center and trial steering committee {5d}

The trial steering committee is composed of research assistants, coordinators, nurses, and ophthalmologists and will meet bi-monthly and on an as-needed basis to discuss the advancement of the trial and address any difficulties in participant recruitment.

#### Composition of the data monitoring committee, its role and reporting structure {21a}

A data monitoring committee will not be required for this trial given that it will involve a relatively small number of patients and compare procedures that are already part of the standard of care.

#### Adverse event reporting and harms {22}

All adverse events (defined as a deviation from the ideal postoperative evolution) will be reported in the trial using the novel Classification of Ophthalmological Complications (COC; see Table 1) to be able to capture the severity of all complications.

#### Frequency and plans for auditing trial conduct {23}

The Research Ethics Board (REB) of the CHU de Québec – Université Laval reserves the right to audit the trial conduct in an independent process from the investigators and funders. However, no predetermined audits will be pre-planned.

#### Plans for communicating important protocol amendments to relevant parties (e.g., trial participants, ethical committees) {25}

Important protocol amendments will be obligatorily communicated to the Institutional Review Board of the CHU de Québec – Université Laval and reported in the trial registration on ClinicalTrials.gov. If these amendments pertain to trial participants and affect their participation in the study, these will also be communicated to them.

#### Dissemination plans {31a}

The results of this trial and any secondary analyses will be reported through a presentation in ophthalmology and/or subspecialty retina conferences, as well as publication in ophthalmology and/or subspecialty retina peer-reviewed journals.

## Discussion

This will be the first 2 × 2 factorial RCT examining repair techniques in primary RRD. It will also be the first RCT to compare gas tamponade between the two most common agents. Notably, it will be adequately powered to detect an effect size as that reported in the PRO study if this difference between treatments truly exists. The use of multimodal imaging will also be a novel aspect of this study, allowing us to compare head-to-head the impact of adding an SB to the retina’s recovery after RRD repair and of differing gas tamponades.

Primary RRD affects approximately 6.3 to 17.9 per 100,000 population [15] and can cause important visual impairment if not corrected promptly. Patients who undergo phacoemulsification with intraocular lens implantation for symptomatic cataract are also at greater risk of developing RRD subsequently. The number of patients affected by RRD is therefore expected to increase in the coming years. Until now, the treatment of RRD has been largely guided by pragmatic retrospective cohort studies. There is a lack of strong evidence guiding therapeutic decisions, and this trial will address (1) whether supplemental SB is justified and (2) whether longer duration gas tamponade with C_3_F_8_ is necessary.

## Trial status

Protocol version 4; October 31, 2023. Recruitment start date: September 2023; approximate recruitment end date: July 2027.

### Supplementary Information

**Additional file 1. **Quality of life and vision questionnaires used in the trial at postoperative follow-up.

## Data Availability

The principal investigator and co-investigators will have access to the final trial dataset. This could be made available to other research teams upon reasonable request and after evaluation of the request by the trial team.

## Abbreviations

BCVA: Best-corrected visual acuity
C_3_F_8_: Perfluoropropane
CHU: Centre Hospitalier Universitaire
CI: Confidence interval
COC: Classification of Ophthalmological Complications
ERM: Epiretinal membrane
ETDRS: Early Treatment Diabetic Retinopathy Study
GCL: Ganglion cell layer
OCT: Optical coherence tomography
OCT-A: Optical coherence tomography angiography
PHVA: Pinhole visual acuity
PPV: Pars plana vitrectomy
PPV-SB: Pars plana vitrectomy with scleral buckle
PVR: Proliferative vitreoretinopathy
RD: Retinal detachment
REB: Research ethics board
RNFL: Retinal nerve fiber layer
RPE: Retinal pigment epithelium
RRD: Rhegmatogenous retinal detachment
SB: Scleral buckle
SF_6_: Sulfur hexafluoride
SSAS: Single-surgery anatomic success
