# Supplementary material for: REtinal Detachment Outcomes Study (REDOS): study protocol for a factorial, randomized controlled trial
Source: Trials. 2023 Dec 20;24:820. doi: 10.1186/s13063-023-07815-x (PMC10734075; doi:10.1186/s13063-023-07815-x)
Supplement: Supplementary file 1 — Additional file 1. Quality of life and vision questionnaires used in the trial at postoperative follow-up. [file 13063_2023_7815_MOESM1_ESM.docx]

**REDOS TRIAL – Additional file 1**

Quality of life questions

1. (Postoperative week 2 questionnaire only): From 0 (none) to 10 (intolerable / worse pain in your life), how would you grade the ocular pain you had immediately after the surgery?
2. (Postoperative week 2 questionnaire only): Did you need to take powerful painkillers (narcotics) for your pain? Yes / no
3. From 0 (none) to 10 (intolerable / worse pain in your life), how would you grade the ocular pain you have now?
4. Do you need to use more artificial tears since your surgery? Yes / no
5. Do you need to use more painkillers since your surgery? Yes / no
6. (Postoperative week 2 questionnaire only): From 0 (none) to 10 (intolerable), how would you grade the difficulty in maintaining the head position after the surgery?
7. After how many days could you restart your normal activities?
8. How many days did you need to be on leave-of-absence from work?
9. From 0 (none) to 10 (intolerable), how would you grade your current visual distortion (?

Quality of vision questions

1. Do you see double when you have both eyes open in your day-to-day? Yes / no

I’m going to read you some statements about problems which involve your vision or feelings that you have about your vision condition. After each question I will read you a list of possible answers. Please choose the response that best describes your situation.

Please answer all the questions as if you were wearing your glasses or contact lenses (if any).

Please take as much time as you need to answer each question. All your answers are confidential. In order for this survey to improve our knowledge about vision problems and how they affect your quality of life, your answers must be as accurate as possible. Remember, if you wear glasses or contact lenses for a particular activity, please answer all of the following questions as though you were wearing them.

1. At the present time, would you say your eyesight using both eyes (with glasses or contact lenses, if you wear them) is excellent, good, poor, very poor, or are you completely blind? (Circle One)
   1. Excellent = 1
   2. Good = 2
   3. Fair = 3
   4. Poor = 4
   5. Very Poor = 5
   6. Completely Blind = 6

The next questions are about how much difficulty, if any, you have doing certain activities wearing your glasses or contact lenses if you use them for that activity.

1. How much difficulty do you have reading ordinary print in newspapers? (Circle One)
   1. No difficulty at all = 1
   2. A little difficulty = 2
   3. Moderate difficulty = 3
   4. Extreme difficulty = 4
   5. Stopped doing this because of your eyesight = 5
   6. Stopped doing this for other reasons or not interested in doing this = 6
2. How much difficulty do you have doing work or hobbies that require you to see well up close, such as cooking, sewing, fixing things around the house, or using hand tools? (Circle One)
   1. No difficulty at all = 1
   2. A little difficulty = 2
   3. Moderate difficulty = 3
   4. Extreme difficulty = 4
   5. Stopped doing this because of your eyesight = 5
   6. Stopped doing this for other reasons or not interested in doing this = 6
3. Because of your eyesight, how much difficulty do you have finding something on a crowded shelf? (Circle One)
   1. No difficulty at all = 1
   2. A little difficulty = 2
   3. Moderate difficulty = 3
   4. Extreme difficulty = 4
   5. Stopped doing this because of your eyesight = 5
   6. Stopped doing this for other reasons or not interested in doing this = 6
4. How much difficulty do you have reading street signs or the names of stores? (Circle One)
   1. No difficulty at all = 1
   2. A little difficulty = 2
   3. Moderate difficulty = 3
   4. Extreme difficulty = 4
   5. Stopped doing this because of your eyesight = 5
   6. Stopped doing this for other reasons or not interested in doing this = 6
5. Because of your eyesight, how much difficulty do you have going down steps, stairs, or curbs in dim light or at night? (Circle One)
   1. No difficulty at all = 1
   2. A little difficulty = 2
   3. Moderate difficulty = 3
   4. Extreme difficulty = 4
   5. Stopped doing this because of your eyesight = 5
   6. Stopped doing this for other reasons or not interested in doing this = 6
6. Because of your eyesight, how much difficulty do you have visiting with people in their homes, at parties, or in restaurants? (Circle One)
   1. No difficulty at all = 1
   2. A little difficulty = 2
   3. Moderate difficulty = 3
   4. Extreme difficulty = 4
   5. Stopped doing this because of your eyesight = 5
   6. Stopped doing this for other reasons or not interested in doing this = 6

The next questions are about how things you do may be affected by your vision. For each one, I’d like you to tell me if this is true for you all, most, some, a little, or none of the time.

|  | All of the time | Most of the time | Some of the time | A little of the time | None of the time |
| --- | --- | --- | --- | --- | --- |
| 1. Do you accomplish less than you would like because of your vision? | 1 | 2 | 3 | 4 | 5 |
| 1. Are you limited in how long you can work or do other activities because of your vision? | 1 | 2 | 3 | 4 | 5 |

For each of the following statements, please tell me if it is definitely true, mostly true, mostly false, or definitely false for you or you are not sure. (Circle One On Each Line)

|  | Definitely True | Mostly True | Not Sure | Mostly False | Definitely False |
| --- | --- | --- | --- | --- | --- |
| 1. I stay home most of the time because of my eyesight. | 1 | 2 | 3 | 4 | 5 |
| 1. I have much less control over what I do, because of my eyesight. | 1 | 2 | 3 | 4 | 5 |
| 1. Because of my eyesight, I have to rely too much on what other people tell me. | 1 | 2 | 3 | 4 | 5 |
| 1. I worry about doing things that will embarrass myself or others because of my eyesight | 1 | 2 | 3 | 4 | 5 |

**REFERENCES**

Potic J, Bergin C, Giacuzzo C, Konstantinidis L, Daruich A, Wolfensberger TJ. APPLICATION OF MODIFIED NEI VFQ-25 AFTER RETINAL DETACHMENT TO VISION-RELATED QUALITY OF LIFE. Retina. 2021 Mar 1;41(3):653-660. doi: 10.1097/IAE.0000000000002894. PMID: 32568989; PMCID: PMC7889283.
